# Immunomodulation and Immune Escape Strategies of Gastrointestinal Helminths and Schistosomes

**DOI:** 10.3389/fimmu.2020.572865

**Published:** 2020-09-17

**Authors:** Marie Wiedemann, David Voehringer

**Affiliations:** Department of Infection Biology, University Hospital Erlangen and Friedrich-Alexander University Erlangen-Nuremberg (FAU), Erlangen, Germany

**Keywords:** nematode, trematode, immunomodulation, type 2 immunity, parasites, schistosomes, Helminths

## Abstract

Parasitic worms (helminths) developed various immunoregulatory mechanisms to counteract the immune system of their host. The increasing identification and characterization of helminth-derived factors with strong immune modulatory activity provides novel insights into immune escape strategies of helminths. Such factors might be good targets to enhance anti-helminthic immune responses. In addition, immunosuppressive helminth-derived factors could be useful to develop new therapeutic strategies for treatment of chronic inflammatory conditions. This review will take an in depth look at the effects of immunomodulatory molecules produced by different helminths with a focus on schistosomes and mouse models of hookworm infections.

## Introduction

Helminths are the most commonly found group of parasites in humans. Especially in regions with poor hygiene standards and insufficient access to medical care, helminths are spread easily and, in some cases, cause severe damage to the infected subjects. They are transmitted in various ways, (for e.g., via contaminated food, water or soil, or by close contact to animals). Most helminth infections are easily treatable with common anti-helminthic drugs, however, untreated infections can endanger especially young children or elderly people. Helminths are relatively large multicellular organisms that can cause tissue damage when larval stages migrate through different organs or adult worms feed on host tissue. Yet, the co-evolution of helminths with their hosts established mechanisms that prevent excessive immune responses so that the parasites can persist and complete their life cycles. Helminths produce a range of different, often highly specialized molecules, changing the general microenvironment around them, altering the density of tissue, or influencing certain types of immune cells. The parasites produce various kinds of these immunomodulatory molecules simultaneously in order to support the individual needs of the parasites in their different life stages (1). Many helminths evolved rather complex life cycles, sometimes requiring intermediate hosts to further develop before infecting their targeted final host. Helminths constitute a genetically very diverse group of organisms and can be divided in two major phyla: nematoda and platyhelminthes both of which include species with potent immunomodulatory function that will be discussed below (2).

## Life Cycle of Schistosomes

Schistosomes, also known as blood flukes, belong to the group of trematodes within the phylum Platyhelminthes. Adult schistosomes show a distinct sexual dimorphism, the female worm being much smaller than the male. The male will surround the female, keeping her in his *gynacophoric canal* for the entirety of their lives. The adult schistosomes can live for up to 10 years inside the blood stream, where they will proceed to lay eggs inside of mesenteric veins in the bowel or rectum. From here, the eggs translocate to the liver and are excreted via feces or they reach the bladder and leave the body with the urine, depending on the type of schistosome. In water, secreted eggs hatch into miracidia larvae, which will proceed to enter a snail serving as the intermediate host. Next, they develop successive generations of sporocysts before being released as swimming cercariae larvae. These will now penetrate the skin of humans or other mammals and become schistosomula, entering the blood stream. Then, they migrate to the lung and liver and mature to fully grown adults, eventually finding a mate, and starting the reproductive cycle again.

Infections with schistosomes can cause schistosomiasis, a disease affecting about 200 million people world-wide. The disease is caused by eggs trapped in tissues where they are surrounded by granulomas composed of various cell types to prevent tissue damage by egg-derived enzymes and pro-inflammatory factors. Excessive granuloma formation can lead to tissue fibrosis and organ failure (3).

## Mouse Models of Hookworm Infections

Parasitic nematodes, or roundworms, in humans include filaria, ascarids, trichurids, and hookworms. Many effects of nematodes on their hosts are studied in mice, using for example the rodent parasites *Nippostrongylus brasiliensis* or *Heligmosomoides bakeri* (formerly named *H. polygyrus*). The life cycle of *N. brasiliensis* is very similar to that of *Necator americanus* and *Ancylostoma duodenale*, the two main hookworm species infecting humans. Although *N. brasiliensis* is mainly found in rat populations and considered a “rat hookworm” that can be used to infect mice under laboratory conditions, a recent study showed that this helminth can also be isolated from wild *Mus musculus* in Korea (4). Eggs of *N. brasiliensis* can be found in the soil, where they will hatch to worms and molt twice before they become infective larvae. They will burrow through the skin of their host and enter the venous system. Then, they are transported to the lung, where they reside in the capillaries, molt again, rupture the capillaries, and enter the alveoli, are coughed up and swallowed. In the lumen of the small intestine, the worms molt for the last time to become fully mature. After mating, the female worm starts to lay eggs, which are secreted via the feces. Immunocompetent mice usually expel the adult worms by day 10 after infection by a process that requires IL-4- or IL-13-mediated activation of STAT6 in intestinal epithelial cells and smooth muscle cells to induce goblet cell hyperplasia, mucus secretion, and increased intestinal peristalsis causing a “weep and sweep” mechanism of worm expulsion (5, 6).

In contrast to the *N. brasiliensis* infection model, *H. bakeri* causes a chronic infection of the small intestine. In this model eggs in the soil hatch and develop into L3 stage larvae that are then taken up orally. The larvae are directly transported into the intestine where they burrow in the submucosa of the gut, molt once again and penetrate the muscular layer of the gut, where they develop into adult worms within 7 days. Male and female worms will then enter the intestinal lumen, mate and release eggs, which are excreted with the feces. Depending on the genetic background of mice and the strength of the immune response the adult worms can live for several weeks and continue to produce eggs (5, 6).

## Response of the Immune System to Helminth Infections

**Figure 1:**
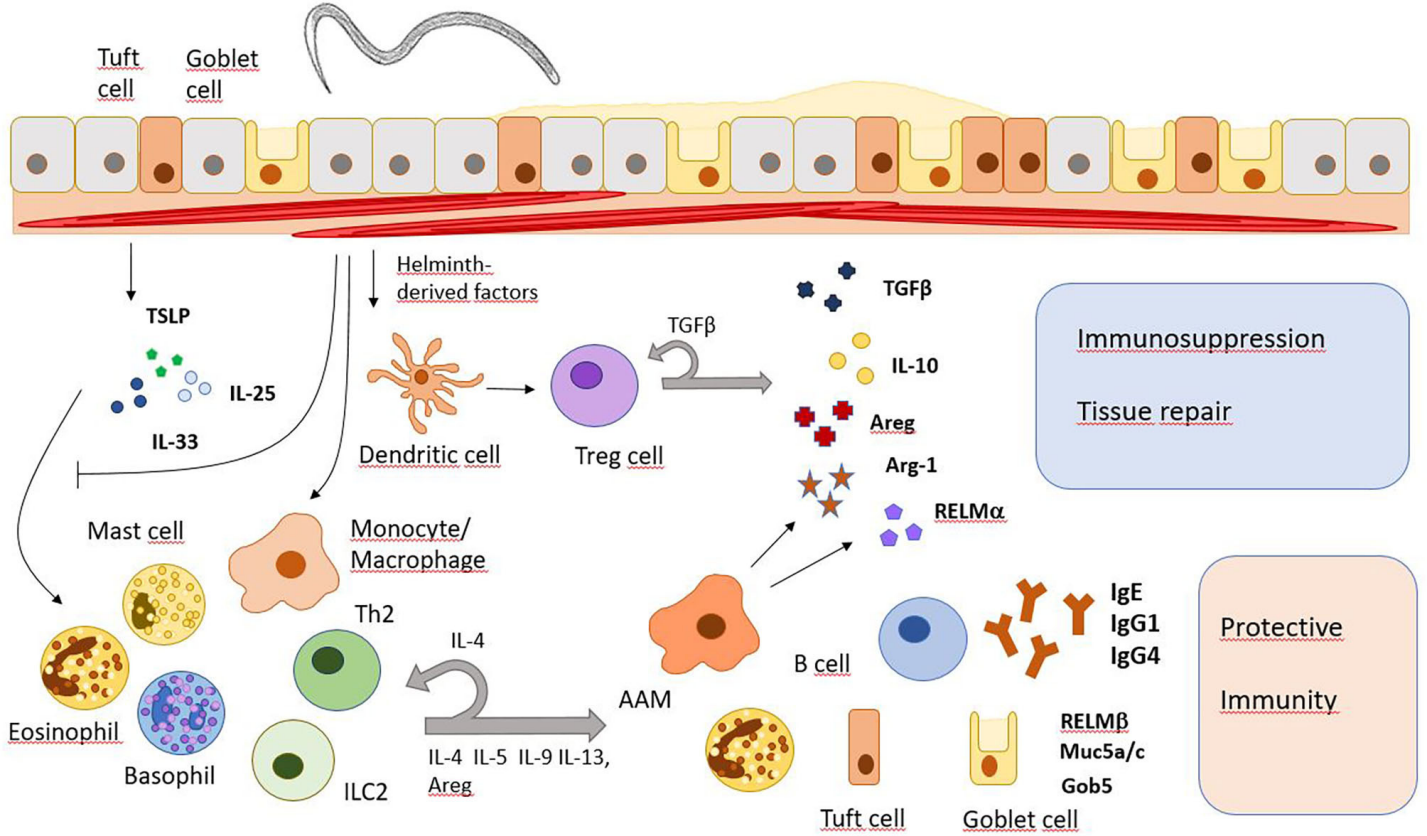
Major immune response pathways after helminth infection. Helminth infections induce the release of alarmins (IL-25, IL-33, TSLP) which subsequently promote immune responses that promote worm expulsion or granuloma formation but also tissue repair and immunosuppression. However, they also inhibit the IL-33 signaling pathway and modulate gene expression in monocytes/macrophages and T cells. Areg, amphiregulin; Arg-1, arginase 1; Gob5, a Calcium-activated chloride channel in goblet cells; Muc5a/c, mucins 5a and c; RELM, resistin-like molecule.

Generally, type 2 immune responses with high IgE levels, increased numbers and/or activity of Th2 cells, type 2 innate lymphoid cells (ILC2), eosinophils, basophils, mast cells, and alternatively activated macrophages (AAMs) are major characteristics of helminth infections (Figure 1). However, since different helminths inhabit distinct niches in the host's body, use different ways of entering the host and show disparate migration within the body, immune responses may vary depending on the infecting helminth species.

Compared to other pathogens, helminths are relatively large and very motile. Tissue damage caused during infection can be sensed by mucosal epithelial cells and keratinocytes. In response, these stromal cells will produce alarmins like interleukin 25 (IL-25), thymic stromal lymphopoietin (TSLP), and IL-33. Next, alarmins induce activation and differentiation of type 2 immune cells which then release several other cytokines like IL-4, IL-5, IL-9, and IL-13. IL-4 and IL-13 activate goblet cells to produce mucus while also triggering smooth muscle cell contractions, the recruitment of eosinophils and the differentiation of AAMs (7). IL-25 is mainly produced by tuft cells in the gut epithelium upon infection with *N. brasiliensis*, leading to activation of Th2 cells and ILC2s, which will start producing IL-13 in response. IL-13 will then lead to extensive differentiation of tuft and goblet cells and therefore, promotes an effective anti-helminthic mechanism (8–10). Later, the Th2 response also drives immunoglobulin class switch recombination in B cells to produce IgE and IgG4 in humans, or IgE and IgG1 in mice, directed mainly by IL-4R/STAT6 signaling and direct T-B cell interaction (11). The IgE antibodies can activate basophils involved in protective immunity during secondary helminth infections (12). Helminths residing in the peripheral blood of their host like *Schistosoma mansoni* also trigger a type 2 immune response during tissue migration and after the release of eggs. Granuloma formation around schistosome eggs is dependent on T cell-derived IL-4/IL-13 secretion and AAMs are critical to prevent a fatal infection in mice (13, 14). Helminth infection further induces the expansion of regulatory T cells (Tregs) and immunoregulatory monocytes along with higher levels of immunosuppressive cytokines IL-10 and TGFβ (15). Therefore, long term helminth infection shows similarities to other chronic infections where downregulation of the immune system prevents extensive harm to the body.

Helminths use several mechanisms of these immunoregulatory functions in order to ensure their own survival and minimize harm to the host caused by tissue damage when migrating through organs. Excreted proteins, metabolites, and extracellular vesicles are used to modulate the infected host's immune response by induction or suppression of immune cells, interfering with signaling pathways and the tissue reparative response to remodel their environmental niche in the host toward their own favor (Table 1). In this review, a selection of potent immunoregulatory factors of certain nematodes as well as schistosomes are described in detail. We will not discuss mechanisms of immunomodulation by helminth-derived exosomes and small RNAs which are covered by other recent reviews (56–58).

**Table 1:** List of helminth-derived factors and their described functions.

**Immunomodulating molecule**		**Parasite/s**	**Effects**	**References**
HpARI		*H. bakeri*	Hinders release of IL-33	(16, 17)
HpBARI		*H. bakeri*	Blocks ST2	(18)
HpBARI_Hom2				
Unknown factor		*H. bakeri*	Stimulates IL-1β production in immune cells	(19)
HpTGM		*H. bakeri*	Ligand for TGFβ receptor	(20–22)
Unknown factor		*H. bakeri*	Decreases Smad7 in FoxP3^−^ IL-10^−^ CD4^+^ T cells; Promotes Treg cell differentiation	(23)
GDH		*H. bakeri*	Induces an anti-inflammatory eicosanoid shift in macrophages	(24)
p43		*T. muris*	Binds IL-13	(25)
PAS-1		*A. suum*	Decreases eosinophilia; Lowers Th2 cytokines; Diminishes IgE; et al.	(26–29)
CPIs	HpCPI	*H. bakeri*	Immunomodulation of DCs	(30)
	AlCPI	*A. lumbricoides*	Affects perivascular infiltrating cells; Influences eosinophils, neutrophils and goblet cells in the lung; Reduces Th2 cytokines; Shift to IgG; Increases Tregs in spleen; Immunomodulation of HmoDCs	(31, 32)
	Nippocystatin	*N. brasiliensis*	Inhibition of T cell proliferation & cytokine production; Decreases IgE level; Inhibits processing by lysosomal cysteine proteases	(33)
	AvCystatin	*A. viteae*	Reduce APC efficiency, T cell response and allergy; Induce regulatory macrophages	(1)
	Onchocystatin	*O. volvulus*		(1)
	SjCystatin	*S. japonicum*.		(1)
AChE		*S. mansoni, S. haematobium, S. bovis, S. japonicum, N. brasiliensis, F. hepatica, D. caninum, et al*.	Motoneuronal function; Alters macrophage response; Influences cytokine production	(34, 35)
Na-ASP2		*Necator americanus*	Involved in tissue migration process; Induces neutrophil and monocyte influx; Supresses B cell receptor signaling	(36–38)
Nb-DNase II		*N. brasiliensis*	Cuts NETs from neutrophils	(39)
smCKBP		*S. mansoni, S. japonicum, S. haematobium*	Influences recruitment of immune cells and granuloma size; Binds certain chemokines	(40, 41)
IPSE/α1		*S. mansoni, S. haematobium, et al*.	Induces IL-4 and IL-13 release from basophils; Induces IL-10 in B cells; Binds IgE	(14, 42–48)
Omega-1		*S. mansoni*	Th2 cell polarization; Drives DCs to promote Th2 cells; Downregulates DC maturation, function, and cytokine production; Enhances IL-1β production in peritoneal macrophages	(49–51)
Unknown factor		*S. mansoni*	Initiates DC driven Th2 cell polarization	(52)
SmSP2		*S. mansoni*	Hinders blood clot formation; Promotes migration, host invasion & immune evasion mechanisms; Processing of nutrients	(47, 53)
Calpain		*S. mansoni and S. mekongi*	Cuts fibronectin	(54, 55)

## Interference With Cytokine Responses by Secreted Factors From *H. BAKERI* and Other Nematodes

Many helminth infections elicit rapid release of IL-33, a member of the IL-1 family. IL-33 has a short-lived role in the early immune response, being released by necrotic epithelial and endothelial cells. It is then quickly oxidized, leaving it inactive (59). It can function as an alarmin to alert the immune system of recent tissue damage or stress. The receptor for IL-33, composed of ST2 (IL-1RL1) and IL-1 receptor accessory protein (IL-1RAcP) is mainly expressed by innate immune cells and Th2 cells, driving the Th2 cell immune response and inducing a strong cytokine production (60). To counteract this response *H. bakeri* releases H. polygyrus alarmin release inhibitor (HpARI). It consists of three complement control protein (CCP) modules, one of which, the N-terminal CCP module pair (CCP1/2) binds to nuclear DNA. The remaining module binds active IL-33, attaching it to the DNA, hindering its release from necrotic cells and therefore, preventing interaction with its receptors on immune cells. Studies have shown that HpARI interferes with the mode of action of both human and mouse IL-33. It was proven that HpARI successfully reduced the eosinophilic response following *N. brasiliensis* infection and increased the worm burden significantly. It was also shown to weaken the reaction of ILC2s in an allergy related model (16, 17). *H. bakeri* further secretes two factors named HpBARI and HpBARI_Hom2 which also contain CCP modules and directly block the IL-33 receptor ST2 (18). However, this is not the only way *H. bakeri* is able to diminish the IL-33 as well as the IL-25 response. It is also capable of inducing the production of IL-1β, mainly in regions with inflammatory cells in the peritoneum and the intestine (19). Here, levels are especially high in areas with a high parasite burden. The source of IL-1β are multiple cell types, nonetheless, CD11b^+^ macrophages seem to be the main generators in an infection with helminths. Yet to be identified *H. bakeri* products stimulate IL-1β production in immune cells via the NLRP3 inflammasome pathway and NFκB activation in an inflammatory environment (19). A receptor for IL-1β, IL-1R1, is predominantly expressed on intestinal epithelial cells. It was proven that IL-1β signaling in a parasitic context leads to a diminished expression of IL-33 and IL-25. Therefore, this mechanism of *H. bakeri* seems to be a very effective self-protective response (19).

Another immunomodulatory mechanism of *H. bakeri* is the activation of the TGFβ receptor on T cells in order to induce the production of Treg cells and various other immunoregulatory immune cells. TGFβ shows a large variety of anti-inflammatory functions. Made for example by dendritic cells (DCs) and Treg cells, it regulates a variety of different immune cells. It inhibits antigen presentation on DCs and macrophages, downregulates the effector functions in macrophages and NK cells, but also increases chemotaxis in eosinophils, mast cells and macrophages. Regarding the adaptive immune response, TGFβ decreases the cytotoxicity in CD8^+^ effector T cells, as well as IFNγ expression and migration of resident memory T cells, while upregulating CD103 integrin. It also promotes the development of Treg cells, Th17 cells, Th9 cells, and IgA producing plasma cells, whereas it inhibits development of Th1 cells, Th2 cells, Th22 cells, and cytotoxic T lymphocytes (CTLs) (61). While some effects of TGFβ can have hostile activity against parasites, the immunoregulatory functions are outweighing the stimulatory effects. Interestingly, *H. bakeri* produces a TGFβ mimic protein (HpTGM) which has no structural homology to TGFβ, but still acts as a ligand for the TGFβ receptor triggering mouse and human FoxP3 expression in T cells (20). HpTGM is made up of five domains distantly related to CCP, and a family of homologs of HpTGM have been found in secretions of *H. bakeri*. However, TGFβ receptor ligand functions have only been proven in some of them (21). Other effects similar to TGFβ function, like promoting production of IL-17 and no induction of Th1 or Th2 cell mechanisms were also shown in HpTGMs (22).

Apart from secreting these TGFβ mimic proteins, *H. bakeri* also promotes TGFβ and IL-10 production by host cells in the gut and decreases Smad7 in FoxP3^−^ IL-10^−^ CD4^+^ T cells. Smad7 inhibits TGFβ signaling by blocking phosphorylation of Smad2/3, the start of the receptor signaling cascade. When Smad7 is downregulated in the CD4^+^ T cells, they respond to TGFβ by differentiation into FoxpP3^+^ and/or IL-10^+^ Treg cells (23). Providing these different mechanisms to enhance TGFβ signaling further promotes the survival of *H. bakeri*.

*H. bakeri* further induces an anti-inflammatory eicosanoid shift in macrophages by secretion of glutamate dehydrogenase (GDH) (24). Macrophages respond to GDH by induced expression of cyclooxygenases and production of prostaglandin E2 (PGE2) which displays some anti-inflammatory functions. This effect appears to be dependent on p38 MAPK activity and requires expression of hypoxia-inducible factor-1 alpha (HIF-1α).

The most abundant secreted protein p43 of the murine whipworm *Trichuris muris* was recently crystallized and identified as IL-13-binding protein which can inhibit IL-13-induced differentiation of alternatively activated macrophages (25).

*Ascaris suum*, the giant roundworm which inhabits the gut of pigs, secretes another protein with strong immunomodulatory function for the host cytokine system. This so-called Protein 1 from *Ascaris suum* (PAS-1) displays anti-inflammatory attributes in the lung and can diminish eosinophilia, decrease Th2 cytokines and lower IgE levels (26, 27). PAS-1 is secreted by larval and adult *A. suum* and apart from reducing eosinophils, it also impairs eosinophil peroxidase activity, and reduces levels of IL-4, IL-5, IL-13, and eotaxin. Additionally, it lowers expression of TNFα, IL-1β, and IL-6 in peritoneal macrophage cultures, increases IL-10 and TGFβ and suppresses influx of neutrophils. The partial induction of regulatory cytokines in macrophages could be one of the main reasons why PAS-1 is decreasing inflammation. Moreover, the anti-inflammatory traits of PAS-1 seemingly depend on the presence of IFNγ and IL-10, suggesting that these factors are necessary for the mode of action of PAS-1 (28). It also diminishes both the level of IgE and IgG1 in the serum of its host (29). Despite these various effects, the molecular mechanism of PAS-1-regulated immune responses remains to be identified.

## Helminth-Derived Cysteine Protease Inhibitors Affect Antigen Presentation

Other main modulators of the immune system produced by nematodes are cystatins, also called cysteine protease inhibitors (CPIs). CPIs effectively inhibit proteases, which are responsible for many immune functions. Antigen recognition by the immune system is dependent on cleavage of foreign proteins to be displayed as peptide antigens in MHC II on the surface of antigen presenting cells (APCs). By inhibiting the responsible proteases for this process, parasites gain another possibility of modulating the immune response. CPIs are detected in many different parasites and show a wide variety of effects on many immune cells. For example, *H. bakeri* CPI (HpCPI) displays a strong immunomodulatory response in DCs. Bone marrow-derived CD11c^+^ DCs (BMDCs), when treated with HpCPI and stimulated with CpG, a Toll-like receptor 9 ligand, have a lowered expression of CD40, CD86, and MHC class II, while also producing less IL-6 and TNFα (30). Further, CPI treated BMDCs are unable to effectively induce proliferation and IFNγ production in CD4^+^ T cells. Likewise, it was shown that they lead to a weaker antigen-specific antibody response as an untreated control (30). In a mouse model displaying allergic airway inflammation responding to house dust mite, it was shown that CPI from *Ascaris lumbricoides* (AlCPI), one of the most common parasitic worms found in humans, especially affects perivascular infiltrating cells, eosinophils, neutrophils and goblet cells in the lung and reduces Th2 cytokines (31). Also, a distinct shift from IgE antibodies to IgG, mostly IgG2a, was reported. Furthermore, AlCPI leads to an increase in Treg cells in the spleen. *In vitro* tests with human monocyte derived DCs (HmoDCs) treated with AlCPI lead to an immunomodulatory effect, reducing HLA-DR, CD83, and CD86 and inducing IL-10 and IL-6 levels. It also resulted in a stop in HmoDC maturation (32). In a similar fashion, the nematode *N. brasiliensis* expresses the CPI nippocystatin. Tested *in vivo* in an ovalbumin (OVA) immunized mouse model, it proved to inhibit proliferation of OVA-specific T cells and production of cytokines. IgE levels also significantly decreased, whereas IgG1 and IgG2 levels did not decline. *In vitro*, it was proven that processing of OVA by lysosomal cysteine proteases was inhibited by nippocystatin (33). A multitude of other helminths have also evolved to expressing different cystatins, examples are AvCystatin from *Acanthocheilonema viteae*, Onchocystatin from *Onchocerca volvulus*, or SjCystatin from *Schistosoma japonicum*. They exhibit many similarities in their function, like reducing APC efficiency, T cell response and allergic reaction or inducing regulatory macrophages (1). Therefore, expression of cystatins indeed appears as successful parasitic immune escape strategy.

## Helminth-Derived Acetylcholinesterases Modulate the Immune Response

Acetylcholinesterase (AChE) represents a helminth-derived factor which directly interferes with intracellular signaling and gene expression. AChE is expressed in a variety of helminths, where it has been shown to have a broad range of functions. On one side, it is needed to ensure motility, for example in flatworms. Here, AChE controls the communication between acetylcholine (ACh) and nicotinic acetylcholine receptors (nAChR). ACh regulates muscular contraction by membrane depolarization due to ion influx in cells, as its receptor acts as an ion channel. To inhibit this interaction and prevent overstimulation, AChE cleaves ACh to choline and acetate. Because of its important motoneuronal function, AChE is seen as a possible target for drugs against schistosomiasis, because many schistosomes, such as *S. mans*oni, *S. haematobium, S. bovis*, and *S. japonicum* do express AChE. However, a drug targeting AChE, Metrifonate, was not approved due to high toxicity in the host. Further research might validate AChE as a possible vaccine target. AChE was also detected in other helminths besides schistosomes, such as in *N. brasiliensis, Fasciola hepatica*, and *Dipylidium caninum* (34).

Since several types of nAChRs are also present in mammals, executing diverse neurological and muscular signaling functions (62), helminths are also secreting AChE to influence host cell behavior. Transgenic *Trypanosoma musculi*, when expressing an AChE secreted by *N. brasiliensis*, showed a reduced infectivity and lead to splenocytes producing increased amounts of IFNγ and TNFα, while expressing less IL-4, IL-5, and IL-13 (35). This altered cytokine response could explain the observed enhanced macrophage M1 response with diminished arginase-1 activity and increased nitric oxide production. It was suggested that this shift to an M1 immune response might inhibit the induction of AAMs and interfere with expulsion of helminths. It was further shown that macrophages have a cholinergic anti-inflammatory pathway, where signaling with acetylcholine inhibits TNFα release in macrophages (63). The increased TNFα response due to helminth-derived AChE could also reinforce the M1 driven immune response. In mammalian immune cells, AChE is reduced when they encounter large amounts of LPS. A reduced expression results in an increase of ACh and less release of TNFα and several other cytokines. This anti-inflammatory response might protect the body from inflammatory overstimulation (64). Therefore, helminths seem to use the AChE, a factor they already expressed to regulate their muscular functions, and secrete it in order to direct their host's innate immune response away from an anti-helminthic activity.

## Chemokine Mimics and Chemokine Binding Proteins

Ancylostoma secreted protein 2 (ASP2) is produced by different hookworms, including Ancylostoma caninum, Ancylostoma ceylanicum, and Necator americanus. The latter secretes ASP2 (Na-ASP2) during its L3 larval stage. The protein has been considered as a promising target for anti-helminthic vaccines (36). It became of special interest as it is significantly involved in tissue dwelling processes and proteins of this nature are seen as good targets for vaccine strategies. It was proven that Na-ASP2 plays an important role in entry into the host tissue and migration before arriving in the intestine. Specifically, it induces leukocyte influx, mainly composed of neutrophils and monocytes. It is most likely able to act as a chemokine mimic for these cells as it shows similarities in structure and charge to CC-chemokines (37). It might be of question, why a helminth would willingly attract neutrophils, as they are known to trap pathogens. Indeed, skin-penetrating larvae of hookworms cause rapid recruitment of neutrophils which release neutrophil extracellular traps (NETs) trying to immobilize the invaders in the skin. However, the larvae then secrete a deoxyribonuclease (Nb-DNase II) to destroy the NETs enabling them to migrate further to the lung (39). Hookworm larvae may further benefit from edema and local inflammation caused by neutrophils as it increases the permeability of tissues and therefore, enable larval migration with ease through dense tissue of the host. Additionally, an increased amount of neutrophils could decrease contact of the helminth to other immune cells like NK cells or eosinophils, which could cause even more harm to the parasite (37).

However, neutrophil recruitment is not the only effect of Na-ASP2. In a human proteome microarray test, it was seen that Na-ASP2 binds to CD79A, a crucial part of the B cell antigen receptor complex. In human B cells, Na-ASP-2 is able to downregulate the transcription of about 1,000 messenger RNAs (mRNAs) and upregulate around 100 mRNAs. This subsequently leads to a different expression of a multitude of proteins derived from B cells, which notably affect transendothelial migration and suppresses B cell receptor signaling (38).

**Figure 2:**
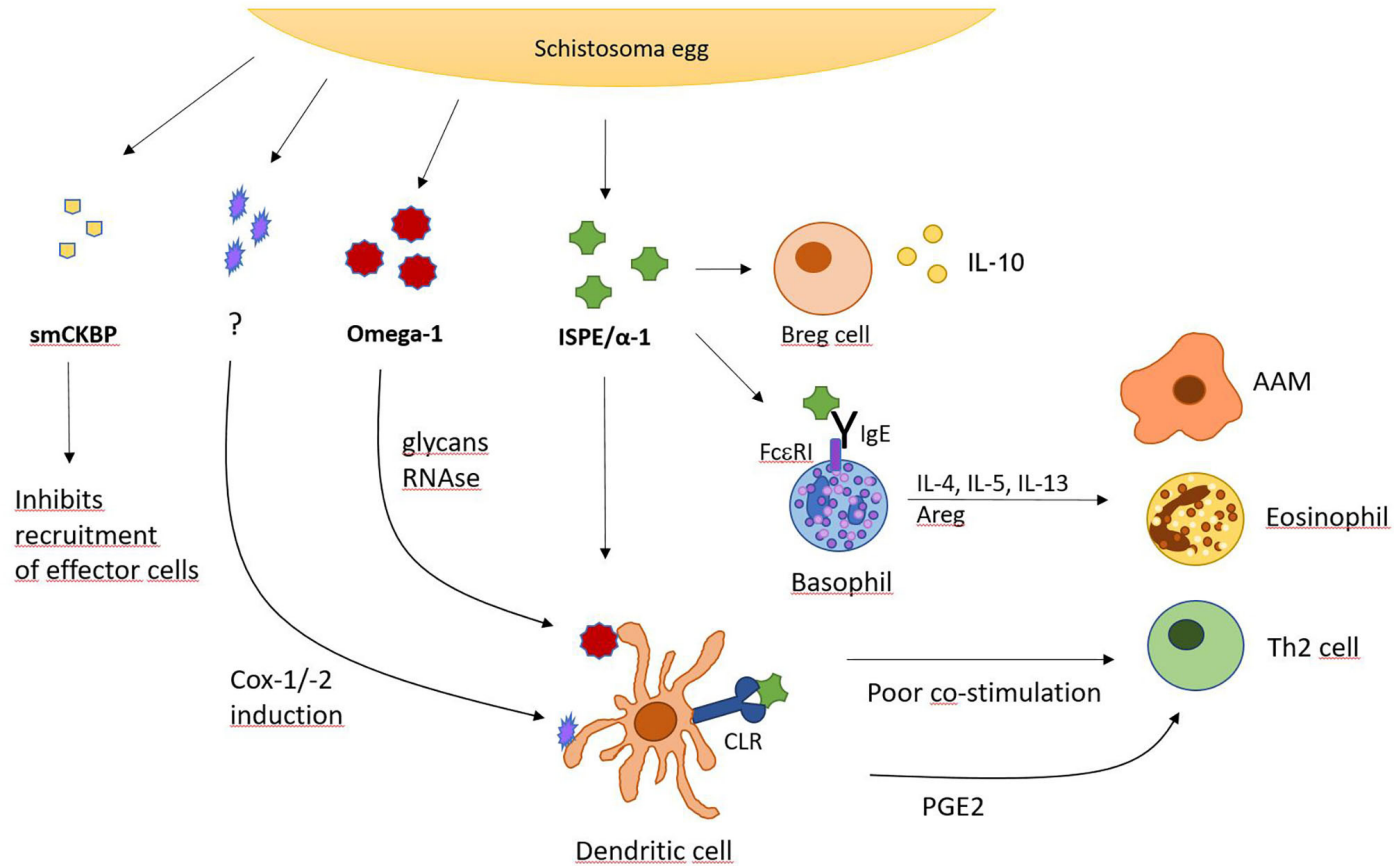
*S. mansoni* egg-derived factors with immunomodulatory activity. Overview of secreted factors from *S. mansoni* eggs and their action on indicated cell types. CLR, C-type lectin receptor; Cox, cyclooxygenase; PGE2, prostaglandin E2.

While parasite larvae require a way to actively migrate through tissue, schistosoma eggs need a possibility to translocate in order to be released from the host's body. Therefore, the blood fluke developed a secretion factor to ensure efficient expulsion of eggs from the host. Surrounded by granulomatous inflammation, many schistosome eggs are trapped in organ tissue, hence, *S. mansoni* eggs have been found to secrete the *S. mansoni* chemokine binding protein (smCKBP), which influences recruitment of immune cells and the size of the surrounding granuloma (Figure 2). Furthermore, smCKBP was also found to be expressed in *S. japonicum* and *S. haematobium*, however, only schistosoma eggs seem to express smCKBP, but none of the other life cycle stages. The smCKBP does interact with a variety of chemokines, including CXCL8, CX3CL1, CCL2, CCL3, and CCL5. By binding to these chemokines, smCKBP prevents them from interaction with their receptors and by this, inhibits chemokine receptor-mediated migration or activation of cells (40, 41). CXCL8, or IL-8, particularly promotes chemotaxis and degranulation of neutrophils (65), but also influences macrophages and mast cells. CX3CL1 does have a chemoattracting effect on monocytes, NK cells and T cells (66). CCL2 is mainly driving chemotaxis of monocytes, but also other cell types, and can regulate further mechanisms, like cytokine secretion or cell adhesion (67). Since CCL3 and CCL5 do have chemotactic properties as well, it is clear that smCKBP targets mainly chemotaxis of immune cells which are for example involved in building the granuloma. Therefore, the protein weakens the surrounding structure, making expulsion of the egg possible. Even though smCKBP has the ability to bind to chemokines, its primary structure has no similarity to proteins from mammals or viral CKBPs. Another interesting finding about smCKBP is that if mice are immunocompromised and for example do not possess CD4^+^ T cells, *S. mansoni* eggs are not excreted as efficiently. Further, smCKBP does not interfere with CCL11 (also named eotaxin-1), and therefore, does not stop eosinophil infiltration (41).

## Other Immunomodulatory Factors of Schistosomes

### IPSE/α1

Besides smCKBP, schistosomes express other potent immunomodulatory factors such as Interleukin-4-inducing principle from schistosome eggs (IPSE), also named α1. IPSE/α1 has been detected in different schistosome species, including *S. mansoni* and *S. haematobium*, which express homologs of IPSE/α1, showing some dissimilarities for example in immunomodulation and expression in life cycles (42). IPSE/α1 from *S. mansoni* eggs, which has been described quite extensively, strongly induces IL-4 as well as IL-13 in basophils and promotes an antibody response. IPSE/α1 is exclusively expressed in the egg stage and released from the subshell area of the egg where it comes into close proximity with various immune cells. Because it induces IL-4, it is presumably involved in activating the Th2 response that is created by *S. mansoni* infections (43). Also, the IL-4 and IL-13 production from basophils induced by IPSE/α1 diminishes inflammatory cytokine responses. This correlates with the fact, that IL-4 and IL-13 also induce AAMs. When stimulated with LPS and IPSE/α1, a stronger IL-4/IL-13 production is generated in basophils and a reduction of inflammatory cytokines in human monocytes is seen. These monocytes also develop an AAM-like phenotype with elevated CD206 and CD209 (44). If the host is not able to produce IL-4 alone or IL-4 with IL-13, the mortality rate increases as the eggs are not excreted sufficiently, causing endotoxemia. Mice with impaired IL-4 production from T cells also exhibit lower granuloma formation and suffer from cachexia (14).

Additionally, it was seen that IPSE/α1 induces IL-10 in naïve B cells. For recombinant IPSE/α1 produced in tobacco plants, it was also shown to be able to induce IL-10 production in human CD1d^+^ B cells. Consistent with these findings, IPSE/α1 is capable of promoting human and murine Breg cells and induce IL-10 production here as well, which results in a reduction of inflammation. The increased level of IL-10 promotes Treg cell development, shifting the host's immune response to a downregulated phenotype. It was also proven in this context, that the development of Breg cells in the marginal zone was not dependent on macrophages, however, it was increased via CD40 ligation (45).

Furthermore, IPSE/α1 binds to IgE via an antigen-unspecific mechanism. This has led to the assumption that it is activating basophils by cross-linking IgE bound to the high-affinity IgE receptor on the cell surface, resulting in release of histamine and activation of cytokine production in Th2 cells (46). However, it was also proposed that IPSE/α1 instead acts via a cross-linking independent mechanism (47). More research might be required to answer these pending questions. Nevertheless, IPSE/α1 does not only act as an extracellular signaling factor but is internalized by certain cell types. It contains a nuclear localization signal (NLS) at its C terminus, which ensures that IPSE/α1 is translocated into the nucleus. When expressed with an enhanced green fluorescent protein (eGFP), extracellular IPSE/α1-eGFP was taken up by CHO cells and transported to the nucleus. This process depends on the presence of the NLS. Human primary monocyte-derived DCs are able to internalize glycosylated IPSE/α1 as well. This mechanism appeared to be dependent on calcium and temperature levels. IPSE/α1 might also directly bind to DNA, regulate gene expression in immune cells and therefore, alter the immune response to the parasite's favor.

A likely way of the host cells to take up IPSE/α1 could be via C-type lectin receptors. These receptors bind to carbohydrates and are expressed on many cell types, including immune cells. IPSE/α1 is glycosylated with two N-glycan sites which are expressing Lewis X motifs (48). Given these circumstances, it is reasonable to speculate that IPSE/α1 is internalized by DCs or macrophages via the C-type lectin receptor pathway but not by basophils which express less C-type lectin receptors (68). Alternatively, DCs and macrophages may take up IPSE/α1 by Fc receptors when antibodies have bound to IPSE/α1. Basophils rather get activated via an IgE-dependent mechanism. In conclusion, IPSE/α1 from *S. mansoni* eggs is a perfect example of how variable a single immunomodulatory protein can be in ways of cell entry and effects on immune cells. However, more research is required to fully understand the impact of this molecule on the host's immune response.

### Omega-1

Another important immunomodulatory glycoprotein produced by *S. mansoni* eggs is Omega-1. This molecule is one of the most abundant proteins in *S. mansoni* egg antigen (SEA) preparations with powerful influence on its host. It is able to drive DCs to a Th2 cell polarization phenotype and hence, beginning of egg production by adult flukes is associated with onset of Th2 development. Th2 cell polarization is usually supported by many different cell types like mast cells, basophils, and eosinophils, which produce IL-4 and other cytokines to induce and promote differentiation and survival of Th2 cells. Apart from these innate immune cells, APCs, especially DCs are also potent drivers of the Th2 response. They capture antigens shed from *S. mansoni* eggs and omega-1 was shown to instruct DCs to prime naive CD4+ T cells to develop into Th2 cells. It can alter and downregulate maturation and function of DCs and cytokine production. This is also shown in DCs treated with LPS, a glycolipid that drives maturation of DCs, as the presence of omega-1 impaired LPS-induced upregulation of CD83 and CD86 on the cell surface (49). Both CD83 and CD86 are important co-stimulatory molecules required for efficient T cell activation.

Furthermore, omega-1 not only stimulates maturation of Th2 cells, but acts as an initiating factor in Th2 differentiation as it promotes acute production of IL-4 *in vivo*. However, it was seen that the omega-1-induced Th2 response does not depend on IL-4 signaling, despite it being a potent Th2 cell driving cytokine (49). Moreover, omega-1 shows RNase activity as well as glycosylation (50). It was shown by site-directed mutagenesis that both do play an important role in Th2 cell polarization via DCs stimulated by omega-1. If either the RNase function or the glycosylation of omega-1 are impaired, DCs are not effectively conditioned to prime a Th2 cell response (50). The DCs take up omega-1 by binding to its glycans via mannose receptors on the cell surface making this internalization process highly dependent on the glycosylation pattern of omega-1. Once the protein is taken up, it degrades ribosomal and messenger RNA and therefore disrupts protein synthesis, conditioning DCs to prime Th2 reactions. In addition, Lewis-X glycan motifs were described on omega-1 which could also polarize Th2 cells (50).

Omega-1 also enhances the production of IL-1β in peritoneal macrophages when stimulated with TLR2 ligands (51). This does not happen with splenic macrophages or DCs. IL-1β is produced when the macrophage receives a signal, for example via TLR2, which triggers NFκB activation and transcription of pro-IL-1β. It requires a second signal, for example by Dectin-1 to form the inflammasome composed of caspase-8 and the ASC inflammasome adaptor protein (also named Pycard). The inflammasome will cleave pro-IL-1β and it will be released from the macrophage. It was shown that omega-1 is able to upregulate inflammasome activity and therefore, increase IL-1β production. The upregulation of IL-1β via inflammasome requires the presence of C-type lectin receptor Dectin-1, ASC, and caspase-8 (51). This showcases the ability of omega-1 to influence multiple pattern recognition receptor pathways and clearly demonstrates once again the multifarious ways a single factor from helminths can influence the immune system.

However, recent research has also discovered an omega-1-independent mechanism on how SEA drives DCs to prime Th2 cells through Dectin-1 and−2 signaling (52). While the exact factor responsible for this second mechanism remains unknown, it was seen that SEA promoted prostaglandin E2 (PGE2) production upon activating Dectin-1 and−2. Activation is followed by a signaling cascade including spleen tyrosine kinase (Syk), extracellular signal-regulated kinase (Erk), cytosolic phospholipase A2 (cPLA2) and cyclooxygenases 1 and 2 (Cox-1, Cox-2). Production of PGE2 leads to expression of OX40 ligand and this subsequently allows DCs to induce Th2 responses (52). Therefore, omega-1 is not the only schistosome-derived factor able to initiate DC-regulated Th2 activation.

### SmSP2

Blood flukes not only need to influence the behavior of immune cells and ensure egg expulsion. Adult schistosomes are of large size and should hence alter normal blood flow and damage endothelial cells around them. This would normally lead to platelet activation and ultimately to the formation of blood clots. Despite this, blood coagulation is hardly ever observed around schistosomes residing in the host's blood vessels. Several mechanisms of schistosomes to hinder blood clot formation have been proposed and one of them is the production of serine protease SmSP2 (69). It consists of three domains: a serine protease domain, a thrombospondin type 1 repeat (TSR-1) and a histidine stretch. SmSP2 orthologs are also found in larval cestodes, *Echinococcus granulosus*, and *Taenia solium*. In adult *S. mansoni* SmSP2 can be found in the tegument and in the excretory/secretory products. It is present at the interface between host and parasite, promotes many different mechanisms in the parasite's migration, invasion of the host and immune evasion, while also being involved in the processing of nutrients. This omnipresence could render it as a potent target for anti-helminthic drugs.

To hinder blood coagulation in its host which would cause not only harm to the parasite itself, rendering it immobile, but also to the host, SmSP2 is especially present in the secreted products. Here, it shows different effects on proteins involved in blood clotting and even regulates the vascular tone. For example, it inactivates vasopressin, a hormone responsible for vasoconstriction leading to increased blood pressure. It also promotes fibrinolysis by activating plasminogen to plasmin, a host protein cleaving fibrin in coagulated blood. It further enhances the production of bradykinin from the host's endothelium, a protein that leads to release of tissue plasminogen activator (tPA). tPA cuts plasminogen to plasmin as well, further reducing blood clots. Additionally, it splits tPA into its more active double chain form, causing even more increase of plasmin. Furthermore, SmSP2 degrades fibronectin in blood clots and the TSR-1 domain in SmSP2 is capable of controlling cell adhesion, which basically allows interaction with other proteins, binding of glycosaminoglycans and inhibits angiogenesis near the schistosome (69). SmSP2 is therefore another clear example of how various the effects of one produced immunomodulatory factor can be. It is also especially interesting to see how diverse the expression pattern of modulatory molecules is between schistosome eggs and adults and that other parasites use the same molecule in different life stages. However, further research is needed on the effect of SmSP2 orthologs in other parasites. Additionally, there are other helminthic proteins showing similarities to SmSP2 in the way they affect the host. In this way, schistosomes possess several other mechanisms to hinder blood clot formation. For example, *S. mansoni* and *S. mekongi* express a tegumental calpain which is capable of cutting fibronectin similar to SmSP2 (54, 55).

Many serine proteases produced by helminths are quite well-described and showcase multiple modes of action. Besides influencing blood coagulation, they also influence the intra- and extracellular metabolism, regulate development and even digestion. As already shown with a few examples in this review, they can influence the composition of the host's tissue, alter cell invasion and help evade the immune system. These characteristics make them probably the most versatile immunomodulatory factors helminths present in the fight against the hosts immune system (53). Many serine proteases would be worth taking a closer look at but describing them would go beyond the scope of this review.

## Discussion

The co-evolution of helminths and their hosts led to an extremely broad range of mechanisms by which helminths influence the immune system of their hosts. They modulate migration, activity, cytokine production and differentiation of innate and adaptive immune cells. The mechanisms to influence the cells are just as diverse as the effects on the immune system in general. The parasites produce proteases to cleave or otherwise influence ligands to hinder activation, secrete adaptors that bind to receptors on the cell surface and influence intracellular signaling cascades. They induce mucus production, promote epithelial cell turnover in mucosal tissues and alter cellular responses. They secrete proteins similar to cytokines or chemokines, inhibit a variety of host factors and can influence the host's RNAs, lowering their expression or cutting them to render them useless for the host.

Consistent with the general notion that most helminths elicit type 2 immune responses, many studies mentioned in this review showed that helminths promote the activation and proliferation of Th2 cells and AAMs. These cell types can be involved in an anti-inflammatory response to infections or tissue damage. Nonetheless, Th2 cells are also described as immune effector cells that promote worm expulsion. Therefore, it might seem strange for helminths to strongly enhance the Th2 cell response. However, it was shown that a short initial Th2 response can contribute to worm expulsion, while long-term activation of type 2 immunity may lead to a shift of the T cell pool to increase the number of Treg cells (70). This is a natural reaction of the host's body to hinder overstimulation of the immune system and therefore, excessive collateral tissue damage in chronic inflammation. The helminths seem to use this response to lower inflammatory reactions and protect themselves from the immune cells, but in the same way also protect the host from tissue damage, even leading to a tissue repairing phenotype. This might be especially useful to the host when the parasite is migrating through tissue to reach different organs.

Distinct macrophage responses also seem to be favored by helminths. This can be explained particularly by AAMs producing anti-inflammatory molecules and helping with clearance of cell debris to avoid a disproportionate recruitment of immune cells. Especially when parasites are damaging tissue due to their large size or when migrating, destroyed cells release alarmins that will attract inflammatory immune cells. Clearance of these alarmins by AAMs helps contain the inflammation but also protects the worm and the host from unnecessary tissue damage. Most sources are consistent with these explanations, but nevertheless, there have also been reports stating that for example the AAM response is unsafe for helminths and instead, the parasite seeks to inhibit AAM production and accepts the production of classically activated macrophages (71). In fact, it has been shown that AAMs protect mice from secondary infections with *H. bakeri* (72) and *N. brasiliensis* (73). Nonetheless, more research is required to understand how AAMs regulate anti-helminth immunity and restore tissue integrity.

The immunosuppressive activity of some helminths can also affect other immune reactions and, for example, ameliorate allergic responses. Hookworm infections can have protective properties against asthma and alleviate atopy caused by some allergens (74). Helminth infections also lead to decreased severity in skin prick tests. On the other side, some helminths, like *A. lumbricoides*, can significantly increase the risk for developing asthma (75). In some instances, helminth infection also worsened allergic responses in the clinical outcome and increased prevalence on aeroallergen-specific IgE. This response could be due to helminth-elicited IgE with cross-reactivity to allergens. Interestingly, many allergens share similarities to secretory products from helminths (76). Epidemiological studies have been performed in countries with poor sanitary conditions and high prevalence of helminth infections. Overall, the results are very inconsistent. Decreases, increases and no change in allergic outcome were observed in association with helminth infections (77). Many other reasons have to be taken into consideration when trying to explain the rise of allergies in developing countries over the past several years, like modernization of life style and a shift to consumption of more highly processed food items. Taken together, helminths seem to be partially able to ameliorate allergic reactions, but numerous studies also show a negative effect on allergic outcome. Because the effects are unpredictable and complex, more investigation on the connection between helminth infections and allergy should be done in order to potentially develop new anti-allergic therapeutics.

Given this immunomodulatory capacity of helminths, it is reasonable to assume that they may interfere with efficient vaccinations against pathogenic bacteria and viruses. Most vaccination strategies rely on the activation of a strong Th1 response along with proliferation of vaccine antigen-specific antibody-producing plasma cells. Since helminths interfere with the host's immune response, some vaccinations seem to be less efficient when administered to a helminth infected patient. When helminth infected mice are administered an influenza virus vaccine, protection against challenge infection, which would normally be provided by the vaccine, is not given anymore. This loss of protection is even seen after the helminth infection is cleared from the mice (78). A similar effect was seen in *S. mansoni* infected persons, where vaccination against tetanus toxoid showed altered immune responses in stimulated peripheral blood mononuclear cells (79). Therefore, preventing helminth infections may help to improve vaccination efficiencies. This could be achieved by raising the hygiene standards and by developing anti-helminthic vaccines which are not yet available for any of the human helminth infections. However, there is an ongoing search for potent vaccine candidates and vaccination strategies (80, 81). Administered at a young age, anti-helminthic vaccines would prevent infections and all negative consequences caused by the infection. Although there are many anti-helminthic drugs available, they have to be administered regularly, as the patients are very likely to become reinfected with parasites once treatment is completed. Thus, a vaccine would be the go-to strategy to ensure long-term protection. A recombinant vaccine against cestodes in livestock has shown efficacy and more vaccines are being developed and tested constantly (82). Despite these accomplishments, further research must be done in order to create an effective, easily producible vaccine approved for use in humans.

## Conclusions

Helminths in general show many similarities in the way they influence the immune system or the way the immune system reacts to them. The most prominent responses are the activation of Th2 cells and AAMs, followed by activation of regulatory T cells and the induction of IgE and IgG4. However, the mechanisms used to influence the immune response are surprisingly diverse. While some helminths seem to have developed modulatory molecules directly against immune functions during thousands of years of co-evolution, others might just have had a role in the metabolism or in the motor function of the parasite. Nevertheless, helminths secrete an extremely broad spectrum of immunoregulatory molecules in order to ensure survival inside their host. The influence of these molecules is not only restricted to the immediate environment of the helminth. It can have consequences in the entire body, influencing for example allergic reactions and the efficacy of vaccinations. Clearly, more research is required in order to completely understand the fascinating world of molecular mechanisms used by parasites and the true impact they have on their hosts.

## Author Contributions

MW and DV wrote the manuscript and designed the figures. All authors contributed to the article and approved the submitted version.

## Conflict of Interest

The authors declare that the research was conducted in the absence of any commercial or financial relationships that could be construed as a potential conflict of interest.

## Abbreviations

AAM: alternatively activated macrophages
AChE: Acetylcholine esterase
CCP: complement control protein
CKBP: chemokine-binding protein
CPI: Cysteine protease inhibitor
HpARI: Heligmosomoides polygyrus alarmin release inhibitor
HpTGM: Heligmosomoides polygyrus TGF beta mimic protein
ISPE: IL-4-inducing principle from schistosoma eggs
PAS-1: Protein 1 from Ascaris suum
SEA: Schistosoma egg antigen
TSLP: thymic stromal lymphopoetin.
